# An Unexpected Case of Severe Arrhythmogenic Right Ventricular Cardiomyopathy

**DOI:** 10.7759/cureus.54922

**Published:** 2024-02-26

**Authors:** Maggie Wassouf, Noura W Masri, Waseem Wassouf, Mohamad J Mansour, Samer R Nasr

**Affiliations:** 1 Internal Medicine, University of Balamand, Beirut, LBN; 2 Division of Cardiology, University of Balamand, Beirut, LBN; 3 General Medicine, American University of Beirut, Beirut, LBN; 4 Non-invasive Cardiology Division, Cardiovascular Imaging, Clemenceau Medical Center, Beirut, LBN; 5 Cardiology, Mount Lebanon Hospital, Beirut, LBN

**Keywords:** cardiac mri, sudden cardiac death, t-wave inversions, near syncope, arrhythmogenic right ventricular cardiomyopathy (arvc/d), arrhythmogenic cardiomyopathy

## Abstract

We present the case of a previously healthy 14-year-old boy who experienced two episodes of lightheadedness while sitting under the sun. The patient did not experience syncope and denied experiencing any other symptoms. Moreover, he exhibited great functional capacity. An electrocardiogram showed T-wave inversions in leads V1 to V4. Subsequent echocardiogram and cardiac magnetic resonance imaging confirmed the diagnosis of arrhythmogenic cardiomyopathy with severe features. Arrhythmogenic cardiomyopathy is a disorder characterized by fibrofatty degeneration of the myocardium and is a common cause of sudden cardiac death. This case highlights the significance of early investigation in any child who presents with seemingly benign symptoms, as they may be indicative of a serious cardiac disease.

## Introduction

Arrhythmogenic right ventricular cardiomyopathy/dysplasia (ARVC/D) is a heritable, progressive disorder that is a well-recognized cause of sudden cardiac death among the young population [1]. It is characterized by fibrofatty replacement of the myocardium and in most cases is associated with a defect in desmosomal genes. The prevalence is approximately 1 in 1,000 to 1 in 5,000 [2]. Although the term ARVC may imply a sole involvement of the right ventricle, some patients exhibit left ventricle-dominant arrhythmogenic cardiomyopathy or biventricular involvement. Thus, the term arrhythmogenic cardiomyopathy was introduced to cover both ends of the spectrum [3]. Presenting symptoms can range from palpitations to arrhythmias causing syncope or sudden cardiac death. There is no gold standard test for the diagnosis of ARVC but rather a combination of clinical tests to meet Task Force Criteria which were revised in 2010. Here, we present the case of a previously healthy 14-year-old male who experienced two episodes of lightheadedness and was found to have T-wave inversions on an electrocardiogram prompting further investigation via echocardiogram (ECG) and cardiac magnetic resonance imaging (MRI). The patient was subsequently diagnosed with ARVC.

## Case presentation

A 14-year-old male with no previous medical or surgical history presented for evaluation after two episodes of lightheadedness one week and one month before the presentation. The patient experienced both episodes while sitting under the sun for a prolonged period and they resolved with two minutes of lying down. He denied any loss of consciousness, palpitations, diaphoresis, nausea, sensation of warmth, weakness, vertigo, or blurry vision during or preceding the episodes. A review of the systems was negative. The patient regularly participated in sports without any complaints or limitations. The patient’s parents denied a family history of premature coronary artery disease or sudden cardiac death. The patient’s vital signs were as follows: blood pressure of 121/70 mmHg, heart rate of 85 beats per minute, and respiratory rate of 18 breaths per minute. The patient was afebrile. Physical examination showed no abnormalities. No dysmorphic physical features were observed.

**Figure 1:**
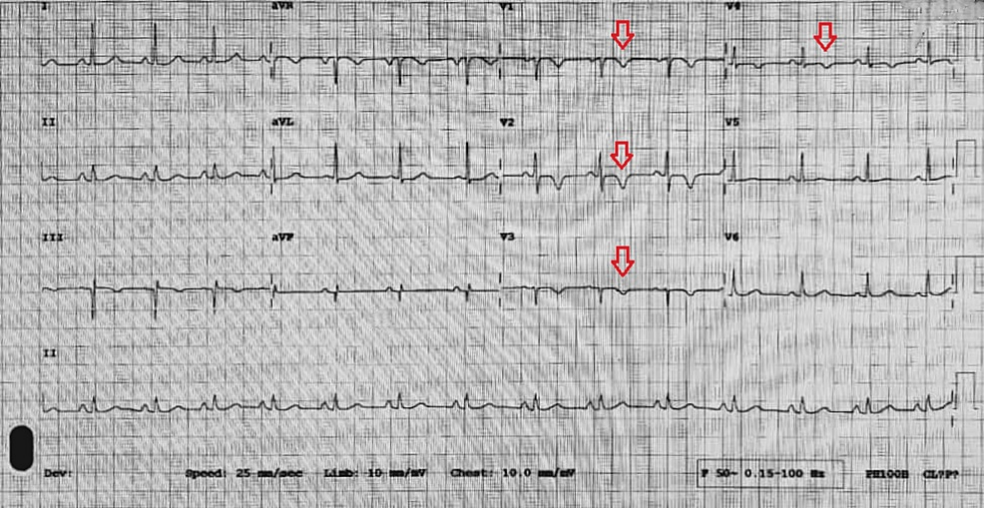
Electrocardiogram showing T-wave inversions in leads V1-V4.

An electrocardiogram (ECG) was performed (Figure 1) and showed T-wave inversions in leads V1 to V4.

**Figure 2:**
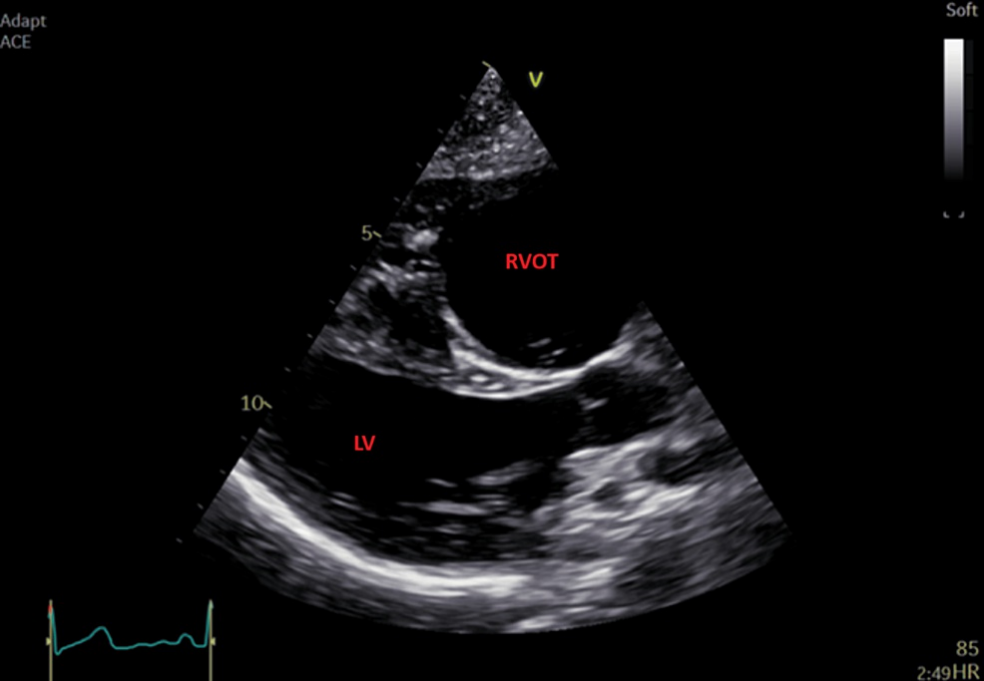
Parasternal long-axis view on TTE showing dilated RVOT. RVOT: right ventricular outflow tract; LV: left ventricle; TTE: transthoracic echocardiogram

**Figure 3:**
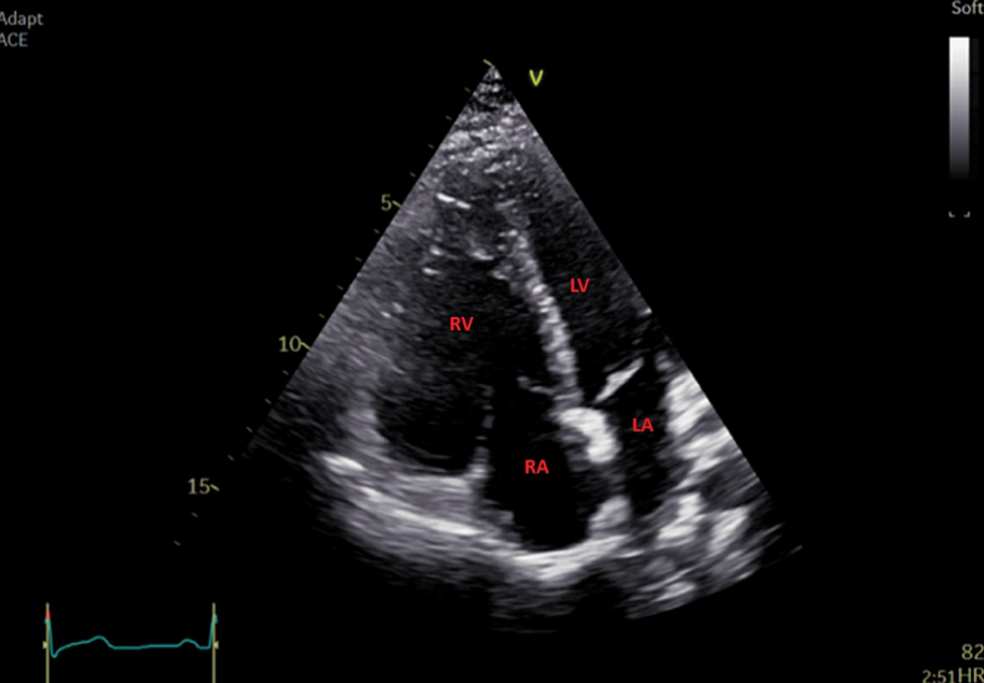
Modified apical four-chamber view on TTE showing dilated right ventricle. RV: right ventricle; RA: right atrium; LV: left ventricle; LA: left atrium; TTE: transthoracic echocardiogram

**Figure 4:**
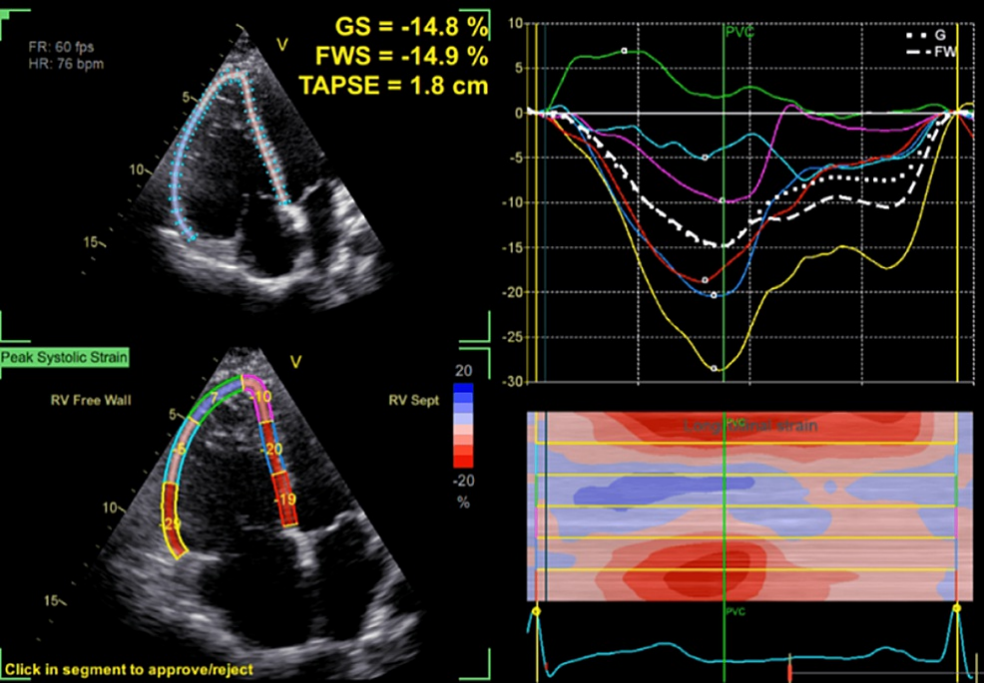
Speckle tracking echocardiography in modified apical four-chamber view showing abnormal right ventricular free wall and global strain values at -15%, with evidence of dyskinesia at the RV apex and positive regional strain of 7%. GS: global longitudinal strain; FWS: free wall strain; TAPSE: tricuspid annular plane systolic excursion; RV: right ventricle

A transthoracic echocardiogram (TTE) was performed and showed a moderately dilated right ventricular outflow tract (RVOT) (44 mm) in the parasternal long axis, mildly dilated right ventricle (RV) at the base (49 mm), and mildly impaired RV free wall strain at -15% with evidence of dyskinetic RV apex. TTE findings are shown in Figures 2-4. A diagnosis of arrhythmogenic cardiomyopathy was suspected requiring cardiac MRI to confirm the suspected diagnosis.

**Figure 5:**
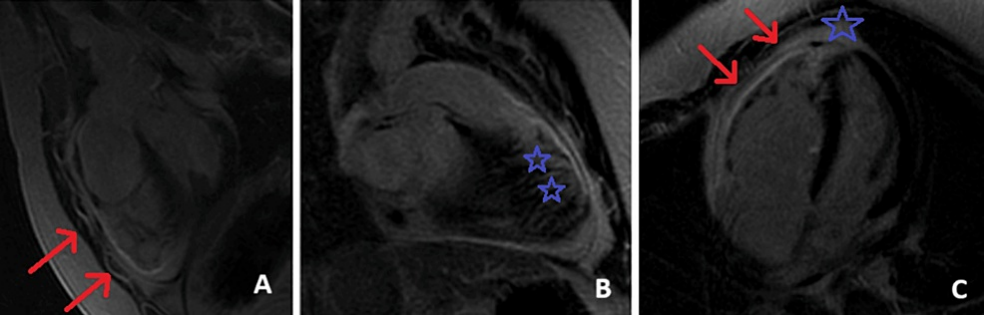
Late gadolinium enhancement sequences showing RVOT views (A and B) with replacement fibrosis (red arrows and blue asterisks) and four-chamber view (C) with fibrosis involving the right ventricular apex (blue asterisk) and free wall (red arrowheads). RVOT: right ventricular outflow tract

Cardiac magnetic resonance (CMR) showed a mildly dilated RV (end-diastolic volume 116 mL/m^2^, 60-108 mL/m^2^), with moderate systolic dysfunction (RVEF 31%). There were four visualized dyskinetic segments of the RV apex, RVOT, and mid-anterior wall. The RV moderator band was prominent. The mid and apical free wall segments of the RV appeared undulated on axial cine-imaging. Delayed-enhancement imaging revealed fibrous replacement of the right ventricle myocardium at the apex, the mid and apical RV free wall segments, and the mid-RVOT segment. CMR findings are shown in Figure 5.

No other fibrosis was present to suggest prior myocarditis, ischemic cardiomyopathy, or infiltrative disease. No RVOT obstruction and no significant pulmonary or tricuspid valve disease (mild tricuspid regurgitation) were present. The pulmonary arteries were not dilated and the right atrium size was normal. Left ventricular size and systolic function were normal. Findings matched major criteria for arrhythmogenic right ventricular cardiomyopathy by cardiovascular MRI.

## Discussion

ARVC/D is a familial disorder marked by the gradual replacement of the ventricular myocardium with fibrofatty scar tissue. This condition increases the likelihood of ventricular arrhythmias and is an important cause of sudden cardiac death [1].

ARVC is a disease of young patients and usually exhibits an autosomal dominant inheritance. Although both genders should theoretically be affected equally, clinically, males are three times more affected than females [2].

ARVC has a broad range of clinical manifestations. Symptoms include lightheadedness, palpitations, or syncope caused by ventricular arrhythmias. Advanced cases present with heart failure symptoms such as dyspnea on exertion and volume overload [4]. Our patient demonstrated symptoms on the lighter end of the spectrum despite having a notable decrease in cardiac function.

The classical form of ARVC typically has four distinct phases. The first phase is an occult phase when the patient is asymptomatic and may or may not have discrete structural abnormalities in the RV but can still present with sudden cardiac arrest. The second phase is the arrhythmic phase when the patient may have palpitations, syncope, and symptomatic ventricular arrhythmias triggered by physical effort. The third phase is right ventricular failure due to the progression of fibrofatty replacement. The final stages have bi-ventricular failure [5].

Diagnosing ARVC is challenging due to the lack of a definitive gold standard test. As a result, diagnosis mainly relies on a qualitative scoring system proposed by the International Task Force in 2010. The evaluation includes non-invasive tests such as ECG, echocardiogram, CMR, 24-hour Holter, and genetic analysis, but invasive tests such as right ventriculography and endomyocardial biopsy are reserved for high-risk disease [5]. One of the major diagnostic criteria is repolarization abnormalities on ECG, namely, T-wave inversion in leads V1, V2, V3, or beyond. Our patient had inversions in leads V1 to V4. Structural abnormalities demonstrated on both TTE and CMR met another major diagnostic criterion.

The management of ARVC focuses on the prevention of sudden cardiac death. The placement of an implantable cardioverter defibrillator (ICD) is the sole intervention that has demonstrated a mortality benefit in the management of ARVC. Other treatment approaches, including medications such as beta-blockers, antiarrhythmics, radiofrequency ablation, and exercise limitation, are used for alleviating symptoms. ICD placement is necessary for patients who experience hemodynamically unstable ventricular arrhythmias and those with LV dysfunction and severe RV dysfunction. It is recommended in patients experiencing ventricular tachycardia and those with major risk factors. The most commonly used medication is a beta-blocker for arrhythmias associated with ARVC. Medical therapy for heart failure is used when necessary [6]. After a discussion of treatment options and possible risks and benefits, our patient was started on bisoprolol fumarate 2.5 mg daily and had an ICD placed. Limiting strenuous exercise was also recommended to prevent disease progression [2].

## Conclusions

This case highlights the significance of early investigation in any child who presents with seemingly benign symptoms, as they may be indicative of a serious cardiac disease. The 2010 revised Task Force Criteria for ARVC were met. Echocardiography and CMR played an important role in the characterization of myocardial abnormalities typical of ARVC. This patient’s atypical mild symptoms were incongruent with imaging findings. The confirmation of the diagnosis allowed early and appropriate interventions to be initiated for the management of the patient’s symptoms and prevention of sudden cardiac death, the most feared complication.
